# 

**Published:** 1839-08

**Authors:** 


### THE AMERICAN JOURNAL OF DENTAL SCIENCE,

### FOR AUGUST, 1839.

Vol I.—No. II.

EDITED BY

CHAPIN A. HARRIS, M. D.

Baltimore.

ELEAZAR PARMLY, ESQ.

New-York.

PUBLISHING COMMITTEE,
E. PARMLY, | E. BAKER,
S. BROWN.

NEW YORK,

GEORGE ADLARD, 168 BROADWAY,

BALTIMORE,

ARMSTRONG & BERRY.

PRICE---THREE DOLLARS PER ANNUM.
Kelley & Fraetas, Printers,

